# Supplementary material for: Modeling glioblastoma heterogeneity as a dynamic network of cell states
Source: Mol Syst Biol. 2021 Sep 16;17(9):e10105. doi: 10.15252/msb.202010105 (PMC8444284; doi:10.15252/msb.202010105)
Supplement: Supplementary file 5 — Source Data for Figure 3 [file MSB-17-e10105-s001.zip › Figure3A_sourcedata/GSEA_3065/hallmarks_state3.GseaPreranked.1623416274744/HALLMARK_ANDROGEN_RESPONSE.html]

Details for gene set HALLMARK\_ANDROGEN\_RESPONSE[GSEA]

|  || Dataset | state3 |
| Phenotype | NoPhenotypeAvailable |
| Upregulated in class | na\_pos |
| GeneSet | HALLMARK\_ANDROGEN\_RESPONSE |
| Enrichment Score (ES) | 0.41088176 |
| Normalized Enrichment Score (NES) | 1.465012 |
| Nominal p-value | 0.019498607 |
| FDR q-value | 0.07400292 |
| FWER p-Value | 0.283 |
Table: GSEA Results Summary

  

Fig 1: Enrichment plot: HALLMARK\_ANDROGEN\_RESPONSE      
 Profile of the Running ES Score & Positions of GeneSet Members on the Rank Ordered List

  

| PROBE | GENE SYMBOL | GENE\_TITLE | RANK IN GENE LIST | RANK METRIC SCORE | RUNNING ES | CORE ENRICHMENT || 1 | B2M |  |  | 11 | 0.538 | 0.0503 | Yes |
| 2 | LMAN1 |  |  | 21 | 0.496 | 0.0968 | Yes |
| 3 | ABHD2 |  |  | 36 | 0.467 | 0.1399 | Yes |
| 4 | SPCS3 |  |  | 76 | 0.370 | 0.1708 | Yes |
| 5 | HMGCR |  |  | 77 | 0.369 | 0.2062 | Yes |
| 6 | SCD |  |  | 108 | 0.340 | 0.2353 | Yes |
| 7 | ACSL3 |  |  | 130 | 0.319 | 0.2634 | Yes |
| 8 | DHCR24 |  |  | 244 | 0.265 | 0.2757 | Yes |
| 9 | TMEM50A |  |  | 253 | 0.262 | 0.2999 | Yes |
| 10 | HMGCS1 |  |  | 267 | 0.254 | 0.3228 | Yes |
| 11 | XRCC5 |  |  | 301 | 0.242 | 0.3422 | Yes |
| 12 | ITGAV |  |  | 322 | 0.238 | 0.3626 | Yes |
| 13 | FADS1 |  |  | 331 | 0.234 | 0.3842 | Yes |
| 14 | SLC26A2 |  |  | 442 | 0.210 | 0.3916 | Yes |
| 15 | ELOVL5 |  |  | 450 | 0.209 | 0.4109 | Yes |
| 16 | AKAP12 |  |  | 640 | 0.180 | 0.4064 | No |
| 17 | IQGAP2 |  |  | 943 | 0.143 | 0.3853 | No |
| 18 | ACTN1 |  |  | 1079 | 0.132 | 0.3825 | No |
| 19 | PGM3 |  |  | 1133 | 0.127 | 0.3886 | No |
| 20 | LIFR |  |  | 1260 | 0.117 | 0.3853 | No |
| 21 | SLC38A2 |  |  | 1321 | 0.114 | 0.3892 | No |
| 22 | GNAI3 |  |  | 1417 | 0.107 | 0.3886 | No |
| 23 | ABCC4 |  |  | 1550 | 0.101 | 0.3830 | No |
| 24 | ARID5B |  |  | 1833 | 0.085 | 0.3587 | No |
| 25 | APPBP2 |  |  | 1862 | 0.083 | 0.3634 | No |
| 26 | DNAJB9 |  |  | 2115 | 0.071 | 0.3412 | No |
| 27 | NGLY1 |  |  | 2156 | 0.070 | 0.3433 | No |
| 28 | IDI1 |  |  | 2285 | 0.065 | 0.3347 | No |
| 29 | SEC24D |  |  | 2587 | 0.054 | 0.3052 | No |
| 30 | XRCC6 |  |  | 2637 | 0.052 | 0.3046 | No |
| 31 | CDC14B |  |  | 2704 | 0.050 | 0.3018 | No |
| 32 | ELK4 |  |  | 2962 | 0.042 | 0.2762 | No |
| 33 | B4GALT1 |  |  | 3043 | 0.040 | 0.2708 | No |
| 34 | INSIG1 |  |  | 3136 | 0.038 | 0.2638 | No |
| 35 | PIAS1 |  |  | 3211 | 0.035 | 0.2587 | No |
| 36 | NCOA4 |  |  | 4078 | 0.013 | 0.1602 | No |
| 37 | FKBP5 |  |  | 4089 | 0.013 | 0.1603 | No |
| 38 | CAMKK2 |  |  | 4124 | 0.012 | 0.1575 | No |
| 39 | PA2G4 |  |  | 4363 | 0.005 | 0.1306 | No |
| 40 | PDLIM5 |  |  | 4368 | 0.005 | 0.1306 | No |
| 41 | ZBTB10 |  |  | 4501 | 0.001 | 0.1155 | No |
| 42 | PLPP1 |  |  | 4721 | -0.004 | 0.0907 | No |
| 43 | ANKH |  |  | 5050 | -0.012 | 0.0541 | No |
| 44 | RPS6KA3 |  |  | 5318 | -0.019 | 0.0251 | No |
| 45 | UAP1 |  |  | 5859 | -0.035 | -0.0337 | No |
| 46 | GSR |  |  | 5892 | -0.035 | -0.0340 | No |
| 47 | NDRG1 |  |  | 6040 | -0.041 | -0.0470 | No |
| 48 | STK39 |  |  | 6252 | -0.048 | -0.0667 | No |
| 49 | ZMIZ1 |  |  | 6296 | -0.050 | -0.0669 | No |
| 50 | CCND3 |  |  | 6959 | -0.079 | -0.1356 | No |
| 51 | SRP19 |  |  | 7050 | -0.084 | -0.1379 | No |
| 52 | HOMER2 |  |  | 7398 | -0.108 | -0.1675 | No |
| 53 | CCND1 |  |  | 7456 | -0.113 | -0.1633 | No |
| 54 | MYL12A |  |  | 7482 | -0.115 | -0.1551 | No |
| 55 | TSC22D1 |  |  | 7546 | -0.122 | -0.1507 | No |
| 56 | TPD52 |  |  | 7598 | -0.128 | -0.1443 | No |
| 57 | CDK6 |  |  | 7605 | -0.128 | -0.1328 | No |
| 58 | AKT1 |  |  | 7627 | -0.131 | -0.1227 | No |
| 59 | CENPN |  |  | 7756 | -0.147 | -0.1234 | No |
| 60 | RAB4A |  |  | 7974 | -0.180 | -0.1311 | No |
| 61 | VAPA |  |  | 7987 | -0.183 | -0.1150 | No |
| 62 | UBE2J1 |  |  | 7992 | -0.184 | -0.0978 | No |
| 63 | ADRM1 |  |  | 8001 | -0.186 | -0.0810 | No |
| 64 | UBE2I |  |  | 8202 | -0.229 | -0.0821 | No |
| 65 | PMEPA1 |  |  | 8520 | -0.346 | -0.0854 | No |
| 66 | SAT1 |  |  | 8533 | -0.354 | -0.0529 | No |
| 67 | DBI |  |  | 8571 | -0.383 | -0.0205 | No |
| 68 | SMS |  |  | 8624 | -0.430 | 0.0146 | No |
Table: GSEA details [plain text format]

  

Fig 2: HALLMARK\_ANDROGEN\_RESPONSE: Random ES distribution      
 Gene set null distribution of ES for **HALLMARK\_ANDROGEN\_RESPONSE**
